# Systemic Inflammation Marker Alterations in Severe Alopecia Areata Patients Treated with Janus Kinase Inhibitors

**DOI:** 10.3390/jcm15010396

**Published:** 2026-01-05

**Authors:** Gokhan Sahin, Fatma Aydin, Esra Pancar Yuksel

**Affiliations:** Department of Dermatology, Faculty of Medicine, Ondokuz Mayis University, Samsun 55139, Türkiye; faydin@omu.edu.tr (F.A.); esra.pyuksel@omu.edu.tr (E.P.Y.)

**Keywords:** alopecia areata, inflammatory markers, JAK inhibitors, neutrophil-to-lymphocyte ratio, systemic immune-inflammation index

## Abstract

**Background/Objectives**: Alopecia areata is an autoimmune disorder characterized by nonscarring hair loss and systemic immune dysregulation. Hematological indices such as neutrophil-to-lymphocyte ratio (NLR), platelet-to-lymphocyte ratio (PLR), monocyte-to-lymphocyte ratio (MLR), mean platelet volume (MPV), systemic immune-inflammation index (SII), erythrocyte sedimentation rate (ESR), and C-reactive protein (CRP) have been associated with inflammatory activity in dermatologic diseases. This study aimed to compare systemic inflammatory markers among patients with severe and mild alopecia areata and healthy controls, and to explore longitudinal changes in these markers in patients with severe disease who achieved clinical improvement following Janus kinase (JAK) inhibitor therapy. **Methods**: This retrospective cohort study included 129 participants: 43 patients with severe alopecia areata (SALT ≥ 50) treated with JAK inhibitors who achieved documented clinical improvement, 43 patients with mild disease (SALT ≤ 20), and 43 age- and sex-matched healthy controls. Hematological inflammatory markers, including red cell distribution width (RDW), MPV, MLR, NLR, PLR, SII, ESR, and CRP, were compared across groups. In patients with severe disease, longitudinal changes were assessed at baseline, three months after treatment initiation, and at the time of documented clinical improvement. **Results**: MLR, NLR, PLR, SII, and ESR levels were significantly higher in the severe group compared with mild cases and controls, while RDW, MPV, and CRP showed no significant differences. Among patients with severe alopecia areata who achieved clinical improvement following JAK inhibitor therapy, NLR and SII decreased significantly over time. MLR, PLR, and CRP also showed reductions during follow-up, while ESR and RDW remained unchanged. **Conclusions**: Systemic inflammatory markers are elevated in severe alopecia areata compared with mild disease and healthy controls. In patients who achieved clinical improvement with JAK inhibitor therapy, several inflammatory indices demonstrated longitudinal changes. These findings are exploratory and suggest an association between systemic inflammation, disease severity, and clinical improvement rather than definitive predictive biomarkers.

## 1. Introduction

Alopecia areata is a chronic dermatological condition driven by immune system dysregulation. It causes nonscarring hair loss by specifically targeting hair follicles in the anagen phase. While the scalp is the primary site of involvement, alopecia areata may also lead to hair loss in other regions such as the eyebrows, eyelashes, beard, and various parts of the body. At any given time, the condition is seen in about 0.1% of the population, and the estimated lifetime risk is around 2% [1].

The underlying mechanisms of alopecia areata have not been fully clarified. However, current evidence suggests that in genetically predisposed individuals, a breakdown in the immune privilege of the hair follicle allows T cells to attack follicular structures [2]. This immune privilege collapse is associated with increased local levels of IFN-gamma and IL-15, and activation of natural killer (NK) cells. IFN-gamma enhances MHC-I expression in follicular cells, promoting antigen presentation to T cells, while IL-15 supports the expansion of NK and T cells and inhibits regulatory T cell function. Both cytokines activate immune responses via the JAK-STAT signaling pathway, which is supported by the clinical benefits observed with JAK inhibitor treatments [3].

Systemic inflammation in the body is commonly indicated by alterations in acute phase reactants, particularly C-reactive protein (CRP) and erythrocyte sedimentation rate (ESR), which are among the most frequently utilized laboratory markers [4]. Red blood cell distribution width (RDW), an indicator of anisocytosis, reflects variations in erythrocyte size and has been associated with systemic inflammation through its correlation with conventional inflammatory markers [5]. Mean platelet volume (MPV), routinely included in complete blood counts, reflects platelet activation and has been increasingly studied as a potential inflammatory indicator [6]. The neutrophil-to-lymphocyte ratio (NLR), platelet-to-lymphocyte ratio (PLR), monocyte-to-lymphocyte ratio (MLR), and the systemic immune-inflammation index (SII) have been increasingly recognized as readily available markers for evaluating systemic inflammation across different pathological states [7]. The SII is derived by multiplying the platelet count by the neutrophil-to-lymphocyte ratio, thereby integrating three major hematologic parameters into one composite marker.

These hematological markers—including NLR, PLR, MPV, and SII—have been investigated in several skin diseases such as psoriasis [8,9], hidradenitis suppurativa [10,11], vitiligo [12], and psoriatic arthritis [13], both as indicators of inflammation and potential reflections of disease activity.

Despite growing interest, data on systemic inflammatory markers in alopecia areata, particularly across different disease severities, remain limited. Accordingly, the present study aimed to evaluate and compare inflammatory markers—including RDW, MPV, MLR, NLR, PLR, SII, ESR, and CRP—across patients with severe and mild alopecia areata, as well as healthy control subjects. In addition, we exploratorily assessed longitudinal changes in these markers in patients with severe disease who achieved clinical improvement following JAK inhibitor therapy, defined as at least a 50% reduction in SALT score. To achieve this, inflammatory parameters were measured at three time points: before treatment, at 3 months after treatment initiation, and at the time of documented clinical improvement.

## 2. Materials and Methods

### 2.1. Study Population

Individuals diagnosed with alopecia areata and admitted to the Department of Dermatology and Venereology at Ondokuz Mayis University Faculty of Medicine between January 2015 and December 2024 were enrolled in the study. Demographic, clinical, and laboratory data were obtained from the hospital information management system. This retrospective cohort study included 43 patients with severe alopecia areata (SALT ≥ 50) treated with JAK inhibitors, 43 patients with mild disease (SALT ≤ 20), and 43 healthy controls. The control group consisted of 43 healthy individuals whose blood parameters were evaluated during routine check-ups. The diagnosis of alopecia areata was primarily established through clinical examination, and dermatoscopic evaluation was performed in cases where diagnostic uncertainty existed, in accordance with previously described diagnostic criteria and characteristic dermatoscopic features reported in the literature [14]

### 2.2. Patient Selection and Cohort Assembly

Patients were retrospectively identified from the dermatology outpatient clinic database of Ondokuz Mayis University Faculty of Medicine between January 2015 and December 2024. Patients with severe alopecia areata were eligible if they had a baseline Severity of Alopecia Tool (SALT) score ≥ 50 and received Janus kinase (JAK) inhibitor therapy during the study period. All patients received JAK inhibitor therapy at standard recommended doses, and no dose adjustments or treatment modifications were performed during the study period. The selection of the specific JAK inhibitor reflected real-world clinical practice and was primarily influenced by drug availability and national reimbursement policies. As baricitinib is not reimbursed in our healthcare system, the majority of patients were treated with tofacitinib, while a limited number received baricitinib.

For longitudinal analyses, only patients who had adequate treatment exposure and achieved documented clinical improvement, defined as a ≥50% reduction in SALT score, were included. Patients who discontinued JAK inhibitor therapy early demonstrated insufficient treatment exposure or lacked standardized follow-up assessments were not classified as non-responders and were excluded from longitudinal analyses, as treatment response could not be validly assessed in these cases. Given the extreme imbalance between treatment subgroups (baricitinib, *n* = 2; tofacitinib, *n* = 41), separate subgroup analyses were not performed, and JAK inhibitors were analyzed as a single therapeutic class.

To allow for cross-sectional comparisons, two additional groups were included: patients with mild alopecia areata (SALT ≤ 20) and age- and sex-matched healthy controls. Mild alopecia areata cases were randomly selected from the same institutional database and time period using a computer-generated randomization list. Healthy controls were selected from individuals undergoing routine health examinations at the same institution and had no history of autoimmune, inflammatory, or hematologic disease.

### 2.3. Data Collection and Clinical Assessment

Demographic and clinical data were retrieved from the hospital information management system. For each patient, information on sex, age, disease duration, family history, comorbid conditions, previous treatments for alopecia areata, treatment duration, follow-up period, and adverse effects was recorded. Disease activity was assessed using the Severity of Alopecia Tool (SALT) score. Clinical improvement was assessed by comparing pre- and post-treatment SALT scores calculated from standardized clinical photographs. Patients were categorized into two groups based on SALT scores: mild (≤20) and severe (≥50) [15].

### 2.4. Laboratory Parameters

Laboratory parameters were obtained from routine complete blood count and biochemistry analyses performed in the same institutional laboratory using standardized methods. For all participants, data on age, sex, SALT score, previous and current treatments, and inflammatory markers—including RDW, MPV, MLR, NLR, PLR, SII, ESR, and CRP—were recorded.

Individuals with systemic illnesses, active infections, malignancies, hematologic abnormalities, or pregnancy were excluded.

### 2.5. Ethical Approval

The study protocol was approved by the Ondokuz Mayis University Clinical Research Ethics Committee (OMU KAEK No: 2025/242). All procedures were conducted in accordance with the principles of the Declaration of Helsinki.

### 2.6. Statistical Analysis

Statistical analyses were conducted using SPSS for Windows, version 22.0 (SPSS Inc., Chicago, IL, USA). Descriptive data were expressed as the mean ± standard deviation or median (minimum–maximum), depending on data distribution. The normality of data distribution was assessed using skewness and kurtosis values in conjunction with the Kolmogorov–Smirnov test.

Differences in sex distribution among the groups were analyzed using pairwise Chi-square tests. Comparisons between groups—severe AA pre-treatment (Group 1), mild AA (Group 2), and healthy controls (Group 3)—were made using one-way ANOVA for normally distributed data, and the Kruskal–Wallis H test for non-normal data. Tukey’s test was performed for post hoc analysis following ANOVA, and Tamhane’s T2 was used where variance homogeneity or normality could not be assumed.

To assess differences across three time points—before treatment (Group 1), three months after treatment initiation (Group 4), and at the point of clinical improvement defined as a ≥50% reduction in SALT score (Group 5)—repeated measures ANOVA was applied for variables with normal distribution. The timing of the third assessment (Group 5) was not standardized and varied substantially among patients, reflecting real-world follow-up conditions rather than predefined study intervals. Effect sizes (partial eta squared) were calculated to estimate the magnitude of observed differences. When significant differences were found, post hoc pairwise comparisons were conducted using the Bonferroni correction to account for multiple testing. For variables that did not follow a normal distribution, the Friedman test was applied as a non-parametric alternative. When the Friedman test revealed a statistically significant difference, pairwise comparisons were carried out using the Wilcoxon Signed-Rank Test.

Patients with incomplete medical records or missing laboratory data were excluded from the study to ensure data integrity and reliable statistical analysis. No data imputation was performed.

A *p*-value below 0.05 was accepted as indicating statistical significance.

## 3. Results

The study included 43 patients with severe alopecia areata (20 females, 23 males; mean age at initiation of JAK inhibitor therapy: 28.07 ± 10.437 years; median: 27; range: 11–47) who initially had a SALT score ≥50 and demonstrated more than 50% improvement following JAK inhibitor treatment. Additionally, 43 patients with mild alopecia areata (SALT score between 3 and 15) were enrolled (21 females, 22 males; mean age: 27.81 ± 10.470 years; median: 27; range 11–47). The healthy controls included 43 participants (20 women and 23 men) with a mean age of 28.05 ± 10.37 years (median: 27; range: 11–48). No statistically significant differences were observed between groups regarding age or gender (*p* > 0.05). Among the patients receiving JAK inhibitors, two were treated with baricitinib, while the others received tofacitinib. Patients with systemic illnesses, active infections, malignancies, hematologic abnormalities, pregnancy, or incomplete medical or laboratory data were excluded from the study.

Table 1 summarizes the values of RDW, MPV, MLR, NLR, PLR, SII, CRP, and ESR for three study groups: patients with severe alopecia areata prior to treatment, those with mild alopecia areata, and healthy controls. As shown in the table, there were no significant differences among the groups regarding RDW, MPV, and CRP levels (*p* > 0.05). In contrast, the MLR, NLR, PLR, SII, and ESR levels were significantly elevated in patients with severe alopecia areata compared to both mild cases and healthy individuals (*p* < 0.05). No statistically meaningful differences were noted between the mild alopecia areata group and the control group for these parameters (*p* > 0.05).

Table 2 displays the values of RDW, MPV, MLR, NLR, PLR, SII, CRP, and ESR in patients treated with JAK inhibitors, assessed at three distinct time points: prior to treatment (Group 1), three months after initiating therapy (Group 4), and after achieving clinical improvement, defined as a minimum 50% reduction in SALT score (Group 5). According to Table 2, changes in RDW and ESR values across the three time points were not statistically significant (*p* > 0.05). Although a slight increase was observed in the mean MPV values across the three time points, this change was statistically significant when all three measurements were compared using repeated measures ANOVA (*p* = 0.023). Nevertheless, pairwise post hoc comparisons did not show statistically significant differences between time points: Group 1 vs. Group 4 (*p* = 1.000), Group 4 vs. Group 5 (*p* = 0.192), and Group 1 vs. Group 5 (*p* = 0.050). This discrepancy may be attributed to high overall variance coupled with relatively small differences between pairs, or to the application of multiple comparison corrections—specifically, Bonferroni-adjusted pairwise t-tests—which may have reduced the likelihood of reaching statistical significance. NLR and SII values significantly decreased with treatment, and this reduction was statistically significant in both the overall comparison across the three time points and in pairwise comparisons (*p* < 0.05). A significant decline in MLR and CRP levels was detected between Group 1 and Group 5 (*p* < 0.05), while no statistically meaningful differences were observed between Group 1 and Group 4 or between Group 4 and Group 5 (*p* > 0.05). Regarding PLR values, a reduction was observed with treatment. Although the decrease between Group 1 and Group 4 was not statistically significant (*p* > 0.05), the differences between Group 1 and Group 5, as well as between Group 4 and Group 5, were statistically significant (*p* < 0.05).

## 4. Discussion

Alopecia areata is an autoimmune skin disorder characterized by patchy, non-scarring hair loss and can occur in individuals of any age or gender. Although mild cases may resolve without intervention, severe or recurrent forms are notably resistant to treatment and are associated with substantial psychosocial burden [16]. Due to its autoimmune pathogenesis, alopecia areata has recently drawn attention in studies exploring the role of systemic inflammation and its association with various hematologic and biochemical markers.

Studies evaluating hematological inflammatory markers (such as RDW, MPV, MLR, NLR, and PLR) and acute phase reactants (ESR and CRP) in patients with alopecia areata are limited and have produced inconsistent findings. The observed discrepancies across studies underscore the necessity of methodologically robust investigations to explore the connection between systemic inflammation and alopecia areata and to assess the clinical relevance of these biomarkers.

In a study by Dere and Gündoğdu, no statistically significant differences were observed in PLR, MPV, or NLR values between 135 alopecia areata patients and an equal number of healthy controls [17]. Consistent with these findings, our analysis also revealed no significant differences in any of the inflammatory markers between patients with mild alopecia areata and healthy individuals. The absence of disease severity classification in their patient cohort makes it difficult to determine whether inflammatory marker levels varied across different clinical presentations.

İslamoğlu and Demirbaş examined 105 patients with alopecia areata and 108 healthy individuals, finding that the RDW, MPV, NLR, PLR, and ESR levels did not differ significantly between groups. Only CRP was elevated in the patient group [18]. In their study, the authors reported that 87 of the patients had a SALT score below 25%, 6 patients had scores between 25–49%, 5 patients between 50–74%, 1 patient between 75–99%, and 6 patients had a SALT score of 100%. Since the majority of patients were classified as having mild disease, this may have contributed to the lack of statistically significant differences observed in most inflammatory parameters compared to healthy controls. In our study, no significant differences were found in inflammatory markers—including RDW, MPV, MLR, NLR, PLR, SII, CRP, and ESR—between the mild alopecia areata group and healthy controls. However, patients with severe alopecia areata showed significantly higher levels of MLR, NLR, PLR, SII, and ESR compared to healthy individuals. These results indicate that systemic inflammation tends to be more prominent in severe alopecia areata and suggest that specific hematologic markers may serve as indicators of the inflammatory burden in more advanced stages of the disease.

Aksoy Saraç et al. evaluated 70 alopecia areata patients and 70 healthy controls [19], concluding that elevated PLR and MLR values may be associated with disease risk and could potentially support the assessment of disease severity. In their study, the authors categorized patients as having mild-to-moderate or severe alopecia areata; however, this classification was based on clinical judgment rather than the widely accepted SALT scoring system. While our findings support the elevation of PLR and MLR in patients with severe alopecia areata, we did not observe significant differences in these markers between patients with mild disease and healthy controls. This suggests that their diagnostic utility may be limited in early or less extensive forms of the disease, but they may hold value in assessing severe alopecia areata.

The study by İcim Komurcugil and Karaosmanoğlu focused predominantly on patients with mild alopecia areata (SALT score < 20%) and demonstrated elevated levels of NLR, MPV, and SII compared to controls, with a significant reduction in these markers following intralesional corticosteroid treatment [20]. In contrast to previous studies, our research focused on a clearly defined cohort of patients with severe alopecia areata (SALT > 50%) who received systemic JAK inhibitor therapy. In this group, levels of MLR, NLR, PLR, SII, and ESR were significantly elevated compared to both patients with mild disease and healthy controls. Moreover, inflammatory markers, particularly NLR and SII, significantly decreased with treatment in the severe group. Unlike previous studies, we systematically compared mild and severe disease and monitored marker dynamics across three time points, emphasizing their potential association with disease severity and changes observed alongside clinical improvement in more advanced cases.

Deng et al., in a large-scale investigation, found that patients with alopecia areata exhibited significantly elevated NLR and SII levels compared to healthy controls, whereas the PLR values did not differ significantly between the groups [21]. Interestingly, when disease severity was taken into account, both PLR and SII were found to be significantly elevated in patients with more severe forms of the disease. These findings show partial overlap with the results of our study. Similarly, we observed higher NLR and SII values in the severe alopecia areata group compared to healthy controls. As reported by Deng et al., we similarly observed a significant rise in PLR levels among patients with severe alopecia areata, while mild cases did not show a notable increase.

The reduction in NLR and SII levels observed after JAK inhibitor treatment in patients with severe alopecia areata indicates that these markers may reflect disease severity and changes observed alongside clinical improvement over time. This finding aligns with growing evidence from other immune-mediated dermatologic conditions. In the study conducted by Çelik and Aktaş, a marked reduction in NLR and SII was noted after six months of IL-17 and IL-23 inhibitor treatment in psoriasis patients, although no significant changes were observed in PLR or RDW [22]. These parallel outcomes across two distinct autoimmune diseases reinforce the idea that NLR and SII appear to be sensitive to changes accompanying targeted immunomodulatory treatment and may reflect systemic inflammation more sensitively than other hematologic parameters.

This study has several limitations. Its retrospective, single-center design may limit generalizability, and unmeasured factors such as psychological stress, smoking status, body mass index, or subclinical infections may have influenced inflammatory marker levels. Longitudinal analyses were restricted to patients with severe alopecia areata who achieved documented clinical improvement following JAK inhibitor therapy. Many patients without documented improvement had insufficient treatment exposure or were lost to follow-up, precluding valid classification as true non-responders. This responder-only design may overestimate treatment-associated changes and introduces potential selection and attrition bias. In addition, the timing of the third assessment point was not standardized, resulting in time-related confounding. Due to the limited sample size, responder-only design, and incomplete confounder data, adjusted regression analyses and predictive modeling were not performed. Accordingly, the findings should be interpreted as exploratory and hypothesis-generating.

## 5. Conclusions

In conclusion, systemic inflammatory markers derived from routine blood tests, including NLR, PLR, MLR, SII, and ESR, were significantly elevated in patients with severe alopecia areata compared with those with mild disease and healthy controls. In patients with severe alopecia areata who achieved clinical improvement following JAK inhibitor therapy, several inflammatory indices demonstrated longitudinal changes over time.

These findings suggest an association between systemic inflammation and disease severity and highlight potential changes in inflammatory markers accompanying clinical improvement. However, given the retrospective design, responder-only cohort, and non-standardized follow-up intervals, the results should be interpreted as exploratory. Further prospective studies with standardized assessments and inclusion of both adherent responders and non-responders are needed to clarify the clinical utility of these markers.

## Figures and Tables

**Table 1:** Comparison of inflammatory marker levels among patients with severe alopecia areata (pre-treatment), patients with mild alopecia areata, and healthy controls.

Parameter	Patients with Severe Alopecia Areata (Group 1) *n* = 43 Mean ± SD Median (Min–Max)	Patients with Mild Alopecia Areata (Group 2) *n* = 43 Mean ± SD Median (Min–Max)	Healthy Controls (Group 3) *n* = 43 Mean ± SD Median (Min–Max)	*p* Values, Respectively Between 3 Groups Groups 1 vs. 2 Groups 2 vs. 3 Groups 1 vs. 3
RDW (%)	13.209 ± 1.055 12.9 (11.9–16.4)	12.804 ± 0.932 12.6 (11.5–17.1)	13.125 ± 1.421 12.7 (11.6–17.8)	0.196 ^α^ 0.178 ^β^ 0.525 ^β^ 0.986 ^β^
MPV (fL)	9.774 ± 1.211 9.9 (6.9–11.8)	10.125 ± 1.026 10.2 (7.6–12.5)	9.995 ± 0.998 9.9 (7.8–12.5)	0.318 ^γ^ 0.293 ^λ^ 0.843 ^λ^ 0.612 ^λ^
MLR	0.305 ± 0.156 0.254 (0.113–0.735)	0.220 ± 0.063 0.207 (0.120–0.359)	0.222 ± 0.071 0.213 (0.122–0.447)	**0.004** ^α^ **0.005** ^β^ 0.996 ^β^ **0.008** ^β^
NLR	2.657 ± 0.988 2.548 (1.407–5.039)	1.640 ± 0.557 1.634 (0.731–2.669)	1.588 ± 0.461 1.469 (0.920–2.919)	**<0.001** ^α^ **<0.001** ^β^ 0.952 ^β^ **<0.001** ^β^
PLR	146.667 ± 50.613 132.487 (80.297–287.500)	105.453 ± 27.570 103.358 (39.534–188.953)	118.813 ± 27.589 113.122 (64.400–175.510)	**<0.001** ^α^ **<0.001** ^β^ 0.082 ^β^ **0.007** ^β^
SII	795.676 ± 339.991 741.312 (384.930–1854.352)	431.953 ± 188.223 400.896 (151.317–1014.220)	422.547 ± 139.886 395.469 (207.174–735.588)	**<0.001** ^α^ **<0.001** ^β^ 0.991 ^β^ **<0.001** ^β^
CRP (mg/L)	3.602 ± 4.999 1.600 (1.000–26.500)	2.037 ± 2.048 1.000 (1.000–11.570)	1.928 ± 1.090 1.740 (1.000–4.750)	0.219 ^α^ 0.177 ^β^ 0.986 ^β^ 0.108 ^β^
ESR (mm/hour)	17.744 ± 13.425 13.000 (2.000–57.000)	10.069 ± 6.385 9.000 (1.000–28.000)	10.674 ± 6.795 10.000 (3.000–31.000)	**0.011** ^α^ **0.004** ^β^ 0.965 ^β^ **0.009** ^β^

RDW: red cell distribution width; MPV: mean platelet volume; MLR: monocyte-to-lymphocyte ratio; NLR: neutrophil-to-lymphocyte ratio; PLR: platelet-to-lymphocyte ratio; SII: systemic immune-inflammation index; CRP: C-reactive protein; ESR: erythrocyte sedimentation rate. ^α^: Kruskal–Wallis H Test, ^β^: Post hoc tests Tamhane’s T2 Test, ^γ^: One way Anova, ^λ^: Post hoc tests Tukey. Bold values indicate statistically significant results (*p* < 0.05).

**Table 2:** Alterations in inflammatory marker levels in the JAK inhibitor group before treatment, three months after treatment initiation, and following ≥50% improvement in SALT score.

Parameter	Before Treatment (Group 1) Mean ± SD Median (Min–Max)	3 Months After Treatment Initiation (Group 4) Mean ± SD Median (Min–Max)	Following ≥50% Improvement in SALT Score (Group 5) Mean ± SD Median (Min–Max)	*p* Values, Respectively Between 3 Groups Groups 1 vs. 4 Groups 4 vs. 5 Groups 1 vs. 5
RDW (%)	13.209 ± 1.055 12.9 (11.9–16.4)	13.234 ± 1.483 13.00 (11.3–20.6)	13.134 ± 1.405 12.9 (11.8–20.1)	0.455 ^α^ 0.898 ^β^ 0.427 ^β^ 0.425 ^β^
MPV (fL)	9.774 ± 1.211 9.9 (6.9–11.8)	9.869 ± 1.136 9.9 (6.8–11.2)	10.107 ± 1.132 10.3 (6.9–12.1)	**0.023** ^γ^ 1.000 ^γ^ 0.192 ^γ^ 0.050 ^γ^
MLR	0.305 ± 0.156 0.254 (0.113–0.735)	0.260 ± 0.081 0.262 (0.127–0.562)	0.237 ± 0.074 0.229 (0.137–0.505)	**0.013** ^α^ 0.184 ^β^ 0.078 ^β^ **0.005** ^β^
NLR	2.657 ± 0.988 2.458 (1.407–5.039)	1.982 ± 0.748 1.857 (0.876–3.509)	1.650 ± 0.576 1.512 (0.816–3.394)	**<0.001** ^γ^ **<0.001** ^γ^ **0.006** ^γ^ **<0.001** ^γ^
PLR	146.667 ± 50.613 132.487 (80.297–287.500)	136.751 ± 43.779 136.453 (68.354–257.931)	120.697 ± 37.020 111.666 (59.681–200.000)	**<0.001** ^γ^ 0.136 ^γ^ **0.001** ^γ^ **<0.001** ^γ^
SII	795.676 ± 339.991 741.312 (384.930–1854.352)	582.743 ± 257.921 502.232 (247.092–1272.256)	472.278 ± 207.728 453.915 (162.104–984.260)	**<0.001** ^α^ **<0.001** ^β^ **<0.001** ^β^ **<0.001** ^β^
CRP (mg/L)	3.602 ± 4.999 1.600 (1.000–26.500)	2.814 ± 2.941 1.090 (1.000–13.500)	2.577 ± 4.176 1.000 (1.000–23.000)	**0.024** ^α^ 0.175 ^β^ 0.139 ^β^ **0.033** ^β^
ESR (mm/hour)	17.744 ± 13.425 13.000 (2.000–57.000)	15.930 ± 13.620 12.000 (1.000–65.000)	15.630 ± 14.098 11.000 (1.000–58.000)	0.381 ^γ^ 0.709 ^γ^ 1.000 ^γ^ 0.953 ^γ^

RDW: red cell distribution width; MPV: mean platelet volume; MLR: monocyte-to-lymphocyte ratio; NLR: neutrophil-to-lymphocyte ratio; PLR: platelet-to-lymphocyte ratio; SII: systemic immune-inflammation index; CRP: C-reactive protein; ESR: erythrocyte sedimentation rate. ^α^: Friedman Test, ^β^: Wilcoxon Signed Rank Tests, ^γ^: Repeated measures ANOVA. Bold values indicate statistically significant results (*p* < 0.05).

## Data Availability

The data that support the findings of this study are available from the corresponding author upon reasonable request.

## Abbreviations

The following abbreviations are used in this manuscript:

NLR	Neutrophil-to-lymphocyte ratio
PLR	Platelet-to-lymphocyte ratio
MLR	Monocyte-to-lymphocyte ratio
MPV	Mean platelet volume
SII	Systemic immune-inflammation index
ESR	Erythrocyte sedimentation rate
CRP	C-reactive protein
JAK	Janus kinase
NK	Natural killer
SALT	Severity of Alopecia Tool
